# Infantile-Onset Pompe Disease: Current Therapeutic Landscape, Clinical Challenges, and Future Horizons

**DOI:** 10.7759/cureus.116696

**Published:** 2026-09-21

**Authors:** Karolina Wyzkiewicz, Klaudia Zinkowska, Veronika Kobylianska, Gabriela Najdek, Julia Molenda, Maria Zimon, Monika Czaplicka, Julia Wróbel, Patryk Mroczka, Hanna Wozny

**Affiliations:** 1 Department of Clinical Genetics, Medical University of Lublin, Lublin, POL; 2 Department of Biophysics, Faculty of Medical Sciences, Medical University of Silesia, Zabrze, POL; 3 Department of Pediatric Hematology, Oncology, and Transplantology, Medical University of Lublin, Lublin, POL; 4 Department of Pediatrics, Medical University of Lublin, Lublin, POL; 5 Clinical Department of Cardiac Surgery, Medical University of Lublin, Lublin, POL; 6 Department of General Pediatrics, Independent Public Clinical Hospital No. 1 of the Medical University of Silesia, Zabrze, POL

**Keywords:** beta-2 adrenergic agonist, enzyme replacement therapy, gene therapy, infant-onset pompe disease, substrate reduction therapy

## Abstract

Infantile-onset Pompe disease (IOPD) is a type of Pompe disease (PD) whose typical form presents in the first months of life. It is the most severe form of the PD spectrum. The underlying metabolic disturbance leads to widespread muscle involvement, affecting both cardiac and skeletal tissues, which critically impairs motor and respiratory function. Because of that, without treatment, the disease quickly progresses and leads to death.

The aim of this article is to present the potential of treatment options for IOPD that have been developed in recent years.

Currently, enzyme replacement therapy (ERT) represents the cornerstone of management, aiming to improve cardiac function, support motor development, and extend overall survival. Advanced therapeutic formulations continue to be explored to enhance treatment outcomes. However, conventional ERT presents inherent limitations, including the logistical burden of lifelong intravenous infusions, potential treatment-limiting immune responses, and restricted passage across the blood-brain barrier.

Moreover, the challenges posed by ERT have encouraged the development of alternative and complementary treatment strategies. Therefore, novel approaches are currently being investigated.

## Introduction and background

Pompe disease (PD) is also known as glycogenosis type II or acid maltase deficiency [1-3]. It is a metabolic, autosomal recessive disorder caused by a deficiency of lysosomal alpha-glucosidase (GAA), an enzyme encoded by the *GAA *gene located on chromosome 17 at the 17q25.2-17q25.3 locus [1,4,5]. GAA hydrolyzes the 1,4- and 1,6-glucosidic bonds of glycogen to produce glucose in the lysosomes [5]. As a result, intralysosomal glycogen accumulates in tissues, especially in cardiac, skeletal, and smooth muscle [3-7]. Moreover, PD also causes the accumulation of autophagic material in muscle fibers. The disease disrupts normal intracellular metabolism, including mitochondrial function with oxidative stress activation and/or cytoplasmic glycogen metabolism, and leads to tissue destruction [5]. 

The overall incidence of this illness has been estimated between 1 in 40,000 and 1 in 146,000 [5,8,9]. However, the incidence rate is higher in some ethnic groups and countries like Taiwan (1 in 17,000) and French Guiana (1 in 2,000) [5,9].

There are two types of PD: late-onset and infantile-onset disease [9]. Its severity depends on the amount of residual enzyme activity [4]. Late-onset Pompe disease (LOPD), which is observed in juvenile and adult patients, is characterized by progressive skeletal myopathy and a prolonged course leading to respiratory failure with relative sparing of cardiac muscle [2,7,10]. On the other hand, in the classic infantile-onset Pompe disease (IOPD), the level of GAA enzyme activity is less than 1%, and illness progresses rapidly [4,5]. This form typically presents in the first months of life, with the median age of onset around four months [1,2,6]. It is the most severe form of the PD spectrum, characterized by cardiomyopathy, cardiomegaly, respiratory insufficiency, generalised hypotonia, muscle weakness, macroglossia, feeding difficulties, and failure to thrive [1-4,6,10]. Without treatment, the condition quickly progresses and generally leads to death due to respiratory and cardiac failure before the second year of life [1,3,6,10]. 

Prompt diagnosis is critical to early initiation of enzyme replacement therapy (ERT), because of the better clinical outcomes of treatment before significant muscle damage [8]. The first-line test is a GAA enzyme assay in dried blood spots [5,7]. In some countries, such as the US, Taiwan, and Japan, it is a part of newborn screening. However, this test is not sufficient for a definitive diagnosis and should be confirmed by GAA enzyme assay in at least one of the following: leukocytes/lymphocytes, cultured fibroblasts from skin biopsy or muscle biopsy. In addition, the molecular analysis of the *GAA *gene may be useful for the estimation of prognosis. Therefore, the pathological GAA assay combined with genetic analysis represents the gold standard for PD diagnosis [5].

Our narrative review encompasses the state of knowledge about PD, a rare disease with early mortality. This review aims to summarize recent advances in PD research, describe novel treatment strategies, and outline directions for future research. 

Methodology 

This article was designed as a narrative review aimed at providing an overview of current and emerging therapeutic strategies for IOPD. The review was not intended to be a systematic review. Given the broad scope of the topic and the different stages of development of the therapeutic approaches discussed, evidence from various study designs was considered, including clinical trials, observational studies, case reports, animal studies, and preclinical research. The review focuses on currently available treatments for IOPD as well as emerging therapeutic strategies, including immunomodulatory approaches, gene therapy, substrate reduction therapy (SRT), in utero enzyme replacement therapy, and methods aimed at improving therapeutic delivery to the central nervous system (CNS). 

Literature search and selection

A literature search was conducted using PubMed/MEDLINE and Google Scholar. Relevant publications were identified using combinations of keywords related to IOPD and its treatment. The main search terms included: "infantile-onset Pompe disease", "Pompe disease", "enzyme replacement therapy", "avalglucosidase alfa", "cipaglucosidase alfa", "immune tolerance induction", "CRIM", "gene therapy", "AAV9", "substrate reduction therapy", "glycogen synthase 1", "in utero enzyme replacement therapy", "central nervous system", "blood-brain barrier", "focused ultrasound", "transferrin receptor".

The final literature search was conducted in August 2026. No strict publication date limits were applied. Priority was given to recent publications; however, earlier studies were included when they provided relevant clinical, mechanistic, or historical evidence.

Inclusion and exclusion criteria

Publications on the treatment, therapeutic management, and new and experimental methods for treating IOPD or PD were included. Publications unrelated to the review’s topic, duplicates, and studies that did not contain sufficient information regarding the therapy in question or its outcomes were excluded.

Evidence synthesis

The available data are presented in the form of a narrative synthesis, organized according to specific therapeutic strategies. Results from clinical trials are presented separately from preclinical and experimental data. Greater emphasis was placed on studies involving humans, while animal studies and in vitro studies were used primarily to illustrate mechanisms of action and new therapeutic possibilities. Due to the small number of patients and methodological limitations of the available studies, their results were interpreted with caution.

Limitations of the present data and review

Several limitations concerning both the available evidence and the review methodology should be acknowledged. First, due to the ultra-rare nature and rapid progression of IOPD, the clinical evidence is predominantly derived from small, open-label cohorts, uncontrolled studies, and isolated case reports (such as initial experiences with in utero ERT and early-phase adeno-associated virus serotype 9 (AAV9)gene therapy trials), which inherently restricts statistical power and generalizability. Second, emerging therapeutic approaches - including SRT, receptor-mediated transcytosis via the transferrin receptor, and focused ultrasound (FUS) for blood-brain barrier (BBB)permeabilization - remain largely at preclinical stages or in early phase I/II clinical trials, precluding definitive conclusions regarding their clinical efficacy and safety profiles in humans. Furthermore, longitudinal follow-up across novel modalities remains short; robust data are lacking concerning the long-term durability of transgene expression, late-onset adverse events, the durability of immune tolerance induction, and progressive neurocognitive manifestations in long-term survivors.

## Review

Clinically approved therapies

Enzyme Replacement Therapy: The Foundation of Treatment

Alpha-glucosidase: Alpha-glucosidase (recombinant human acid α-glucosidase, rhGAA) was approved for the treatment of PD in 2006. The introduction of ERT has been associated with improved clinical outcomes and prognosis, particularly in IOPD. Long-term treatment with rhGAA has been shown to lead to significant regression of hypertrophic cardiomyopathy, improvement in organ function, and prolongation of overall survival and survival without the need for mechanical ventilation [5,11-13].

Available data indicate that avalglucosidase alfa may provide greater improvement in exercise capacity, as assessed by the 6-minute walk test, compared with alglucosidase alfa [5].

Cipaglucosidase alfa: Cipaglucosidase alfa, used in combination with miglustat as an enzyme stabilizer, was approved by the European Medicines Agency in 2023. Clinical trial results suggest that treatment with cipaglucosidase alfa and miglustat may be associated with greater improvement in respiratory function, measured as percentage of predicted forced vital capacity, compared with alglucosidase alfa and placebo. Due to the progressive nature of PD and the irreversibility of some organ changes, treatment in patients with the infantile form should be initiated immediately after diagnosis [5,14].

Dosage: The approved dose of rhGAA is 20 mg/kg of body weight, administered every two weeks as an intravenous infusion [5].

In patients with the pediatric form of the disease who do not show improvement while taking a 20 mg/kg dose or whose response to treatment in terms of cardiac, respiratory, and/or motor function is insufficient, an increase in the dose to 40 mg/kg, also administered every two weeks, may be considered, provided that there are no safety concerns [5,15].

Studies also specify the recommended dose of cipaglucosidase alfa, which is 20 mg/kg every 2 weeks. Additionally, it has been tested in combination with miglustat [5]. This dosage is recommended in the summary of product characteristics [16].

In a study of infants with severe PD, treatment with recombinant acid alpha-glucosidase (GAA) significantly improved survival. Compared with the untreated control group, the risk of death was reduced by 99%, while the risk of death or the need for ventilation decreased by 88-92% [17].

Risk of Anti-drug Antibody Formation

infant-onset of Pompe disease patients are divided into two groups based on the ability to produce GAA enzyme (designated as cross-reactive immunological material positive, CRIM+) and lack of this ability (CRIM-). The first group can produce even non-functional enzymes and will still be CRIM+, whereas the second group has two severe GAA variants and cannot form any native GAA enzyme. This fact makes them prone to anti-drug ERT antibody development because of their inability to develop natural immunological tolerance [18-20]. 

Cross-reactive immunological material status with high and sustained anti-rhGAA IgG antibody titers (HSAT) was viewed as a poor prognostic factor in patients affected by IOPD treated with ERT [18]. These HSAT were specified as antibody titers in excess of 12,800 or at least 1:51,200 [18,19]. In addition, CRIM+ presence is not an infallible indicator of low antibody production, as roughly 30% of CRIM-positive patients develop HSAT, subsequently impairing the efficacy of ERT [18]. Over the years, various immunomodulatory approaches have been tested to mitigate the adverse impact of anti-rhGAA IgG antibodies [18].

Current clinical guidelines for CRIM-negative patients recommend inducing immune tolerance (ITI) via a short course of methotrexate, rituximab, and intravenous immunoglobulin (IVIG) [18]. For patients who are CRIM+, ITI with transient low-dose methotrexate has been successful at inducing tolerance [19]. This underscores the critical demand for predictive immunological biomarkers capable of detecting high-risk CRIM+ individuals prior to ERT initiation or spotting early sensitization during treatment [18].

Eradication of HSAT in PD can be achieved by supplementing the standard ITI regimen (rituximab, methotrexate, and IVIG) with plasma cell-directed therapies, such as bortezomib or daratumumab [19].

A decline in anti-rhGAA IgG antibody levels directly correlated with overall clinical progress, including a reduction in left ventricular mass index and urinary glucose tetrasaccharide, enhanced ventilator and enteral independence, as well as improved motor function [19]. Higher doses of rhGAA may be associated with improved clinical outcomes in patients with IOPD, with sustained cardiac benefits observed in patients who respond well to treatment [20].

Experimental therapeutic strategies at the preclinical and clinical research stages

Beta-2 Adrenergic Agonist: Potential Adjunctive Therapies 

Beta-2 adrenergic agonists, particularly albuterol (salbutamol), have been investigated as potential adjunctive therapies to ERT in patients with IOPD, as disease progression may persist despite early initiation of ERT. One proposed mechanism underlying the therapeutic effects of β2-agonists is the upregulation of the cation-independent mannose-6-phosphate receptor (CI-MPR), which is involved in the cellular uptake of recombinant human acid α-glucosidase (rhGAA) and may thereby enhance the efficacy of ERT. In a clinical study, the efficacy and safety of albuterol as an adjunct to ERT were evaluated in 14 children with IOPD aged 2-12 years, whose disease manifestations had developed before 12 months of age and who had previously received ERT. Albuterol was administered at a dose of 12 mg/day for 26 weeks, and its effects were assessed using the 6-minute walk test, the four-stair climb test, the Gross Motor Function Measure-88, speech quality assessment, and selected biochemical parameters. After 26 weeks of treatment, a statistically significant reduction in the median time required to complete the four-stair climb test was observed among the 12 patients able to perform the task, whereas the other assessed parameters demonstrated only modest or inconsistent trends toward improvement. These findings suggest that albuterol may exert beneficial effects on selected aspects of motor function in children with IOPD; however, the magnitude of the observed clinical benefit was limited [21]. 

With regard to safety, albuterol was generally well tolerated; of 57 adverse events reported during the study, 11 were considered potentially treatment-related, including nausea, increased urinary frequency, and tachycardia, and were generally mild and transient. However, the authors emphasized that the study was open-label and involved a small patient cohort; therefore, the findings do not provide definitive evidence of the efficacy of β2-agonists in IOPD and underscore the need for larger, controlled clinical trials [21]. It is also noteworthy that the efficacy of albuterol as an adjunctive therapy has been investigated in LOPD, where a randomized clinical trial of extended-release albuterol in patients receiving stable ERT demonstrated improvements in parameters including supine forced vital capacity, the 6-minute walk test, and the Gross Motor Function Measure-88 scores. However, findings obtained in LOPD cannot be directly extrapolated to IOPD, although they provide additional rationale for further investigation of β2-agonists as potential adjunctive therapies in PD [22].

Gene Therapy

The limitations of ERT have contributed to the development of research into gene therapy as a potential treatment approach, which aims to deliver a functional copy of the GAA gene to cells, enabling long-term endogenous production of the deficient enzyme. Vectors belonging to the family of recombinant adeno-associated viruses (AAV), particularly the AAV9 serotype, have gained particular significance in this regard. Preclinical studies have shown that AAV9 can effectively transduce cardiac and skeletal muscle, as well as reach structures of the CNS. In animal models, gene therapy using AAV has been shown to reduce glycogen accumulation and improve muscle and respiratory function [23,24]. One of the approaches currently under development is the GC301 (rAAV9-coGAA) therapy, which utilizes a recombinant AAV9 vector containing an optimized sequence of the human GAA gene. Unlike ERT, which requires regular administration of an exogenous enzyme, gene therapy aims to induce the production of the enzyme directly by the body’s own cells. Preclinical studies have demonstrated dose-dependent GAA expression and the presence of the vector in target tissues, which laid the groundwork for initiating clinical trials in children with IOPD [25].

An important issue regarding the efficacy of AAV9 therapy is the immune response against the vector. In a study using a mouse model of PD, it was shown that following intravenous administration of GC301, GAA activity increased in the liver, heart, and muscles, confirming effective gene delivery to various tissues. At the same time, the presence of pre-existing neutralizing antibodies against AAV9 limited the vector’s bioavailability and transduction. These results indicate that testing for anti-AAV9 antibodies may be of significant importance in the selection of patients for gene therapy [25].

The next stage in the development of this strategy was the use of GC301 in children with IOPD. The patients showed increased GAA activity, improved cardiac parameters, and progress in motor development. Some of the children achieved the ability to sit, stand, or walk independently. No antibodies against GAA were detected, while respiratory tract infections were among the most commonly observed adverse events. However, due to the very small sample size and the limited follow-up period, these results should be interpreted as preliminary [26]. 

More recent clinical data also suggest the potential for using AAV9 therapy in older children with IOPD who had previously received ERT for an extended period. The results suggest that gene therapy may potentially reduce the need for chronic ERT even in children who have previously received intensive ERT [26].

Substrate Reduction Therapy: A Novel Experimental Treatment

Substrate reduction therapy is a novel experimental treatment for IOPD that relies on reducing muscular glycogen synthesis and accumulation by suppressing glycogen synthase 1 (*GYS1*) gene expression [27,28]. *GYS1 *encodes a muscle isoform of glycogen synthase; its inhibition has been shown to cause a dose-dependent decrease in glycogen synthesis and lysosomal accumulation primarily in skeletal muscle, with a lesser effect in cardiac muscle [27], while also reducing molecular biomarkers associated with PD [29]. On the other hand, SRT does not target *GYS2*, a hepatic isoform, thereby not limiting hepatic glycogen synthesis for body-wide metabolism [28]. This treatment has no observable toxicity, although the long-term effects of muscle glycogen synthase inhibition remain unknown - there are case reports of patients with loss-of-function *GYS1 *mutations with outcomes such as juvenile exercise intolerance, cardiomyopathy, cardiac arrhythmias, and even sudden cardiac arrest [27,30]. SRT is currently used in the treatment of other lysosomal storage diseases, such as Fabry disease, Gaucher disease, and Niemann-Pick disease type C [27].

Currently, there are three known approaches to SRT in the treatment of PD [27,28,31,32].

Antisense oligonucleotides: These are chemically modified oligomers that hybridize to complementary target RNAs in a sequence‐specific manner and induce their degradation [28]. One of them is a phosphorodiamidate morpholino oligonucleotide conjugated to a cell-penetrating peptide, which works through antisense-induced exon 6 skipping of *GYS1 *and premature stop codon usage in the transcript of *GYS1 *and is currently in a phase II clinical trial [27,28,31].

Short interfering ribonucleic acid: This is used in a conjugate with Centyrin® (Aro Biotherapeutics, Philadelphia, USA)** **protein and facilitates gene knockdown [27,32]. 

Selective small-molecule inhibitors of *GYS1*:Selective small-molecule inhibitors of *GYS1 *include MZ101 [27]; MZE001, which has demonstrated efficacy in vitro and in vivo and has undergone safety evaluation in humans [30]; and substituted pyrazoles, which are currently being investigated in a phase I clinical trial [31].

It is important to note that none of the above-mentioned substrate synthesis inhibitors have been approved for the treatment of PD [28]. 

The literature reports state that SRT has a synergistic effect with ERT in reducing both glycogen synthesis and accumulation in murine models of IOPD and human cellular models, normalizing glycogen levels in some muscle beds. This treatment combination could correct cellular pathways associated with PD; therefore, SRT could potentially be used as an add-on to ERT, helping to enhance and prolong treatment effects [27,28]. 

In Utero Enzyme Replacement Therapy 

The initial application of in utero ERT involved a 37-year-old multigravida woman (G12, P4) with a history of three pregnancies lost to CRIM-negative IOPD. The targeted homozygous *GAA *gene mutation NM_000152.5(GAA).525_526del (p.Asn177fs) was identified early in pregnancy via chorionic villus sampling [33].

Under ultrasound guidance, alglucosidase alfa was infused directly into the umbilical vein at a dosage of 20 mg/kg of estimated fetal weight. The prenatal treatment protocol comprised six biweekly administrations spanning from 24 weeks and 5 days to 34 weeks and 5 days of gestation. The patient delivered vaginally at 37 weeks and 4 days of gestation [33].

Postnatally, an ITI protocol was initiated on day 1 of life, following established management guidelines for CRIM-negative IOPD. Maintenance enzyme replacement therapy with alglucosidase alfa commenced on day 4 at 20 mg/kg every two weeks. To maintain clinical response, the dosage was subsequently escalated to 40 mg/kg biweekly at 9.6 months of age and further increased to 40 mg/kg weekly starting at 11.3 months [33].

Creatine kinase activity remained within the normal range throughout the 13-month observation period. No motor deficits were observed. All echocardiographic examinations, including prenatal examinations, were normal. No pathological abnormalities were identified on placental examination [33].

Urinary glucose tetrasaccharide levels decreased rapidly after initiation of postnatal ERT and subsequently remained within the normal range [33]. 

Attempts to Overcome the Blood-Brain Barrier in IOPD

Despite the substantial improvement in survival achieved with ERT and newborn screening, long-term follow-up has revealed possible CNS involvement in some patients with IOPD. A small case series of patients treated with ERT for 12-15 years reported neurological manifestations, including seizures and encephalopathy, with developmental plateauing observed in some individuals [34]. Neuroimaging studies have further identified progressive white matter abnormalities in long-term survivors, including periventricular signal changes, subtle basal ganglia abnormalities, and symmetric involvement of the deep frontoparietal white matter. These observations raise the possibility that CNS involvement may persist despite long-term ERT and underscore the need for further investigation of CNS-directed therapeutic strategies [35]. 

Role of receptor-mediated transcytosis and BBB shuttles: The pathogenesis of skeletal muscle pathology involves lysosomal glycogen accumulation, dysregulated autophagy, impaired calcium homeostasis, increased reactive oxygen species production, and mitochondrial dysfunction. In contrast, the precise mechanisms linking CNS glycogen accumulation to long-term neurological manifestations remain poorly understood [36]. 

One of the major limitations in the treatment of CNS involvement is the BBB, which significantly restricts the penetration of large molecules, including therapeutic enzymes. One potential strategy to overcome this barrier is the use of receptor-mediated transcytosis, whereby therapeutics are transported across BBB endothelial cells via specific receptors. However, the selection of an optimal receptor with adequate selectivity and therapeutic efficacy is critical. Current research has focused in part on the transferrin receptor (TfR), which represents a promising therapeutic target in IOPD. TfR expression is significantly higher in neonates than in adults, which may translate into increased brain availability of TfR-targeted platforms, such as antibody transport vehicles. Early administration of TfR-targeted acid α-glucosidase to newborns could provide broader enzyme distribution, potentially enabling simultaneous targeting of both the muscular and neurological components of the disease. This strategy may potentially limit glycogen accumulation in the CNS and delay or prevent the development of late neurological complications observed in older patients with IOPD [36,37].

Treatment with FUS: Another notable strategy for drug delivery across the BBB is FUS. This approach induces transient, localized increases in BBB permeability through the combined application of acoustic energy and systemically administered microbubbles. Intravenously injected microbubbles oscillate within the acoustic field generated by FUS, exerting mechanical effects on the walls of cerebral microvessels [38]. This acoustic cavitation transiently disrupts endothelial tight junctions, facilitating the non-invasive entry of therapeutics that would otherwise be unable to cross the BBB. Combining FUS with viral vectors, therapeutic enzymes, or engineered fusion proteins may enhance their brain biodistribution and penetration into the brain parenchyma [38,39].

Preliminary clinical trials in various neurological disorders have demonstrated the feasibility and potential safety of FUS-mediated transient BBB opening. However, the application of this technology in IOPD remains investigational, and there are currently insufficient clinical data to establish its efficacy in this disease [38]. On the other hand, other investigative approaches are evaluating the direct administration of viral vectors into the cerebrospinal fluid via the cisterna magna (intracisternal delivery). This approach bypasses the BBB and may potentially increase the availability of therapeutic agents within the CNS [40].

While much of this research remains at the preclinical stage, direct cerebrospinal fluid delivery and FUS-mediated BBB disruption represent promising complementary investigational approaches to overcome the BBB and potentially address CNS pathology in IOPD [38,40].

## Conclusions

Infant-onset Pompe disease requires early and comprehensive therapeutic management, with ERT remaining the cornerstone of treatment. Despite its substantial clinical benefits, the limitations of current therapeutic approaches highlight the need for continued development of strategies aimed at achieving more durable and comprehensive correction of GAA deficiency. Particular emphasis should be placed on approaches designed to reduce the immune response, improve the efficacy of enzyme delivery, and achieve long-term correction of the underlying genetic defect. Gene therapy, SRT, and other investigational approaches represent potential directions for future treatment development; however, their clinical applicability requires further evaluation. 

Central nervous system involvement remains a major therapeutic challenge due to the limited availability of current treatments within the CNS. Further research should therefore also focus on developing effective strategies for delivering therapeutic agents to the CNS. A comprehensive approach to the treatment of IOPD should integrate effective correction of enzyme deficiency with modulation of the immune response, durable correction of the underlying genetic defect, and the prevention and treatment of CNS involvement.
